# A simple algorithm for the offline recalibration of eye-tracking data through best-fitting linear transformation

**DOI:** 10.3758/s13428-014-0544-1

**Published:** 2015-01-01

**Authors:** Miguel A. Vadillo, Chris N. H. Street, Tom Beesley, David R. Shanks

**Affiliations:** 1 University College London, London, UK; 2 Primary Care and Public Health Sciences, King’s College London, Capital House, 42 Weston St., London, SE1 3QD UK; 3 University of British Columbia, Vancouver, BC Canada; 4 University of New South Wales, Sydney, NSW Australia

**Keywords:** Drift correction, Eye-tracking, Recalibration

## Abstract

Poor calibration and inaccurate drift correction can pose severe problems for eye-tracking experiments requiring high levels of accuracy and precision. We describe an algorithm for the offline correction of eye-tracking data. The algorithm conducts a linear transformation of the coordinates of fixations that minimizes the distance between each fixation and its closest stimulus. A simple implementation in MATLAB is also presented. We explore the performance of the correction algorithm under several conditions using simulated and real data, and show that it is particularly likely to improve data quality when many fixations are included in the fitting process.

Eye-trackers are quickly becoming an essential tool in any cognitive psychology laboratory to explore cognitive processes as diverse as reading comprehension (Rayner, Pollatsek, Drieghe, Slattery, & Reichle, 2007), decision making (Orquin & Loose, 2013), associative learning (Beesley & Le Pelley, 2011; Le Pelley, Beesley, & Griffiths, 2011), and medical diagnosis (Krupinski, 2010). However, eye-tracking can be problematic when researchers need to locate participants’ fixations with a high degree of accuracy and precision. According to the specifications offered by their manufacturers, the average systematic error of the most frequently used eye-tracker systems is around 0.5° or less (e.g., SR Research, Inc., 2013b; Tobii Technology AB 2010). However, there are multiple factors that limit the accuracy and precision of eye-movement data, including operating distance from the screen and participants’ ethnicity (Blignaut &Wium, 2014; Holmqvist et al., 2011; Nyström, Andersson, Holmqvist, & van de Weijer, 2013). Even under optimal conditions, the calibration of the eye-tracker tends to degrade over time due to changes in screen illumination, participants’ fatigue, and other factors. As a result, data become increasingly less accurate as the experiment proceeds. Given the negative impact of these factors, it is not surprising that independent tests conducted by researchers reveal average errors larger than those provided by manufacturers, usually around 1° or more across the duration of a study (Hansen & Ji, 2010; Johnson, Liu, Thomas & Spencer, 2007; Komogortsev & Khan, 2008).

Figure 1 shows two examples of common problems faced by researchers using eye-tracking devices to study fixation patterns. The examples are taken from a real experiment conducted in our laboratory in which participants were asked to find a T among a number of similar distractors and respond to its left/right orientation (for a detailed description of the task, see Beesley, Vadillo, Pearson, & Shanks, in press). In the left panel of Fig. 1 the fixations deviate systematically from their closest stimulus. This situation is particularly likely to arise, for example, due to the cumulative drift of a head-mounted eye-tracker across the experiment. Fortunately, this problem can be ameliorated easily by moving the pattern of fixations up or down and left or right until the coordinates of the fixations match the coordinates of their closest stimulus. Several programs and algorithms allow such drift corrections to be conducted either manually or automatically (e.g., SR Research, Inc., 2013a). However, not all calibration problems can be fixed so easily. The right-most panel of Fig. 1 shows a slightly different situation. In this case, the eye-movement data cannot be corrected by just moving the coordinates of the fixations. Instead, the coordinate system of the fixations needs to be stretched to better fit the pattern of stimuli on the screen.

**Fig. 1:**
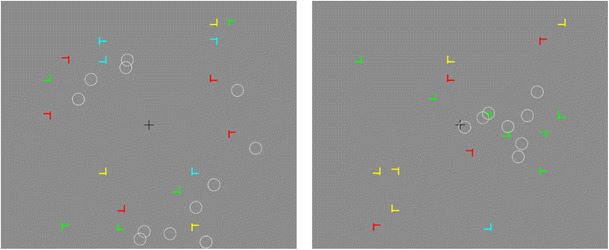
Two examples of common problems found in eye-movement measures. The white circles denote the fixation locations reported by the eye-tracker and the rest of the elements are stimuli presented to the participant. In this particular experiment, the size of each stimulus is 30 pixels square (approx. 0.68° of visual angle)

Given the harmful effect that even small calibration errors can have on the interpretation of experimental data, developing successful and simple methods for the correction of eye-tracking data has quickly become an active area of research in recent years (Blignaut, Holmqvist, Nyström, & Dewhurst, 2012; Buscher, Cutrell, & Morris, 2009; Hornof & Halverson, 2002; Mannan, Ruddock, & Wooding, 1995; Zhang & Hornof, 2011, 2014). For example, a popular method for cleaning up eye-tracking data relies on required fixation locations. If there are areas of the screen where it can be safely concluded that the participant is looking at a specific time, the discrepancy between the coordinates of that area and the coordinates reported by the eye-tracker can be used to correct calibration error. For instance, Hornof and Halverson (2002) used an experimental paradigm in which participants had to click on some items on the screen at the beginning and end of each trial. Assuming that participants were looking at those items when they clicked on them, the disparity between the location of the clicked-on element and the coordinates reported by the eye-tracker could be used to obtain an error signature for each participant. This error signature was then used to correct the gaze-location data. Unfortunately, an important limitation of this approach is that not all experimental paradigms lend themselves to the inclusion of required fixation locations. Sometimes there is no single element in the display where it can be concluded safely that the participant was fixated at a specific moment. Another problem is that this method assumes that the calibration error remains constant across time and trials, an assumption that is unlikely to hold in most cases.

Another popular method for the correction of eye-tracking data is based on measuring the disparity between each fixation and the closest stimulus on the screen (Zhang & Hornof, 2011). In many cases, the magnitude and direction of the disparities is roughly constant for all fixations. Using the procedure described by Zhang and Hornof (2011) it is possible to identify the mode of these disparities and correct all the fixations by adding an error vector that minimizes the distance between the mode of the disparities and the stimuli presented on screen. A potential limitation of this method is that not all calibration errors can be corrected by adding an error vector to the coordinates of all the fixations. For example, the calibration error can be larger in some parts of the screen than others (see, e.g., the data depicted in the right panel of Fig. 1).

The present article explores an alternative procedure for the correction of eye-tracking data. Unlike the procedure described by Hornof and Halverson (2002), the method described below is not based on required fixation locations, but on probable fixation locations. This means that it relies on fixations that are likely (although not absolutely certain) to be directed towards a specific stimulus on the screen. It also makes the procedure ideal for experimental paradigms where the participant cannot be required to fixate on specific items at specific moments. Additionally, the fact that fixations need not be associated with specific target stimuli with absolute certainty means that more fixations can be used in the error-correction process. Any fixation that is more or less directed to any element on the screen, with some degree of error, provides useful information. As described below, under standard conditions, it is possible to use the algorithm with just 5–10 fixations. An advantage of this is that the algorithm can be applied separately to data from different trials, provided that the participant makes at least 5–10 fixations in each trial. Consequently, it is not problematic that the calibration error changes across trials because data from different trials are corrected independently. Unlike the procedure devised by Zhang and Hornof (2011), the present algorithm does not correct fixations by adding a correction vector to all fixation coordinates. Instead, the coordinates of all fixations are corrected by means of matrix-vector multiplications. These linear transformations both move the coordinates of fixations vertically and horizontally to reduce error, as well as stretch or contract the fixation space. This can give rise to larger corrections in some parts of the screen than others.

## Fitting a linear transformation to eye-movement data

The two problems represented in Fig. 1 can be solved by conducting a linear transformation of the vectors coding the coordinates of each fixation. Using linear algebra, this correction can be implemented as a multiplication of two matrices:

1 $$ \mathrm{T}\mathrm{F}=\mathrm{C} $$

where T is a 2 × 2 transformation matrix, F is a 2 × *n* matrix where each column represents the x and y coordinates of one fixation and *n* represents the number of fixations, and C is a 2 × *n* matrix representing the corrected (i.e., transformed) fixation locations, where each column is the linear transformation of its corresponding column in F. Within this mathematical formulation, correcting the eye-movement data simply requires finding an appropriate matrix T that improves the match between the observed fixations represented in F and the actual pattern of stimuli shown to participants. The dimensions of T are 2 × 2, allowing for a mapping of a set of two-dimensional vectors, F, to another set of two-dimensional vectors, C.

Assuming that the participant’s fixations are directed towards stimuli rather than between stimuli (although, as discussed later, this assumption can be relaxed considerably), then it is possible to use the average distance between each fixation and its closest stimulus as a measure of the quality of eye-movement data. Based on this, the optimal T can be defined as the matrix that minimizes the mean distance between each fixation contained in F and the coordinates of the center of its closest stimulus. The best-fitting values of T can be found using any optimization routine such as, for example, the popular Simplex algorithm (Nelder & Mead, 1965).

In ideal conditions, if the eye-movement data reflected perfectly the coordinates of the fixations, then C should be equal to F and, therefore, T should be the identity matrix. Although in reality the calibration of the eye-tracker will rarely be perfect, to the extent that it is reasonably good the T matrix that minimizes the cost function can be expected to be relatively close to the identity matrix. Because of this, the identity matrix can be used as a convenient starting point for the parameter-fitting process.

## A MATLAB implementation

Given that the procedure explained above requires nothing but a series of matrix-matrix multiplications and a simple optimization algorithm, it can be easily implemented in any programming language for scientific computing, such as MATLAB, Octave or R. As an example, Listing 1 shows an implementation of the entire eye-tracking correction process based on a simple MATLAB function.

The first line of code just declares the  function, which takes a set of raw fixation coordinates and a set of stimulus coordinates as input, and outputs the corrected fixation coordinates, stored in .  contains the raw fixation coordinates as a 2 × *n* matrix where each column represents the x and y coordinates of the center of a fixation and *n* refers to the number of fixations in a given trial.  contains the coordinates of the stimuli on the screen in a 2 × *n* matrix where each column represents the x and y coordinates of the center of a stimulus and *n* refers to the number of stimuli.

Line 2 calls the  function from MATLAB’s optimization toolbox. The arguments of the  are the name of the to-be-optimized function, , which is defined below, and the starting point of the optimization process. As explained at the end of the previous section, the identity matrix (no transformation), here defined as , is a convenient starting point. The output of the function, , contains a 2 × 2 transformation matrix that best improves the fit between fixations and stimuli. Line 3 simply corrects the original fixations by multiplying the optimal transformation matrix, , by the raw fixation coordinates matrix, . The final coordinates are stored in , the main output of the  function.

The core of the process is the subfunction , defined in Lines 4–15. This subfunction computes the average distance between each fixation and its closest stimulus given a transformation matrix. The transformation matrix is represented by , which is the only argument of . However, MATLAB subfunctions inherit access to the variables declared in their parent function. This allows  to obtain access to the fixation coordinates and the stimuli coordinates, represented by  and , respectively. Line 5 computes the new coordinates, , that result from the fixation coordinates multiplied by the transformation matrix. Lines 7–13 loop through each fixation in  and through each pair or coordinates in  to calculate the distance between each fixation and its closest stimulus. Line 10 computes the distance between fixations and stimuli using MATLAB’s  function to determine the length of the difference vector. Once the distance between each fixation and its closest stimulus has been computed, line 14 averages the values for all fixations and stores this value in , which is the output of the subfunction .

## When to use it: Exploring some boundary conditions

As explained above, this algorithm relies on the assumption that participants look directly at the center of stimuli appearing on the screen. However, *sensu stricto,* this assumption is hardly ever met in any psychological experiment. Even if participants tend to look at stimuli, they might not look right at their centers. Moreover, there is no reason why participants cannot look at parts of the screen where no stimulus is presented. In fact, in certain contexts it might even be optimal to look at the gaps between stimuli (e.g., Reingold, Charness, Pomplun, & Stampe, 2001). For this reason, in real experiments the algorithm is unlikely to return the exact coordinates of the fixations. In some cases, the algorithm might even distort the real coordinates by, for example, dragging the coordinates too close to the center of the stimuli, which in fact were not the focus of attention. In order to explore the prevalence and impact of these problems, we conducted a series of simulations in which several aspects of representative eye-tracking data were manipulated.

**Fig. 2:**
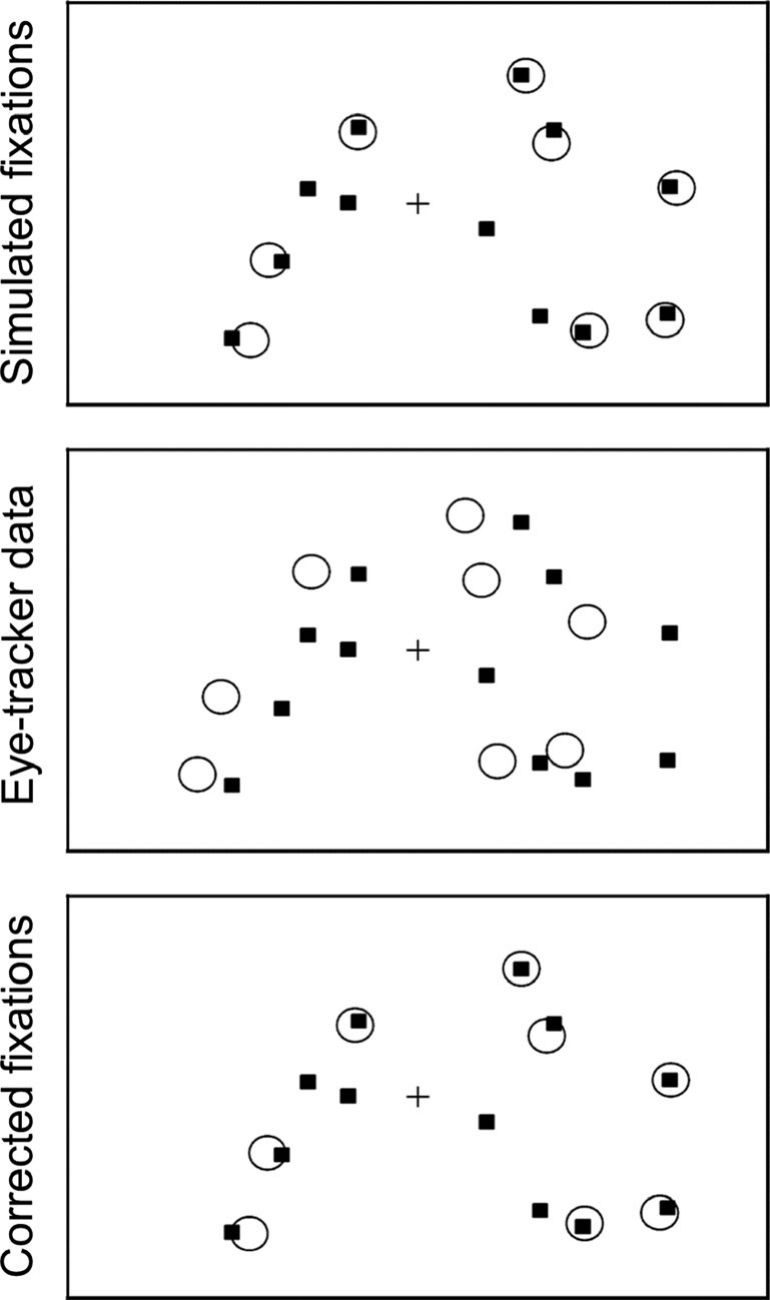
Example of the procedure used in the simulations. Circles depict fixation locations and filled squares depict stimulus items. The top panel represents the real pattern of fixations. The central panel represents how a poorly calibrated eye-tracker would record those fixations. The bottom panel represents the fixations after correcting the simulated eye-tracker data with our algorithm

Figure 2 summarizes the procedure that we used to run our simulations. In each simulation, we first generated a number of stimuli in random locations of a fictitious 1920 × 1200 screen. Stimuli are represented as black squares in Fig. 2. Then, we generated fixations to a subset of those stimuli. These fixations are represented as circles in the top panel of Fig. 2. As can be seen, the coordinates of the fixations did not match perfectly with the centers of their corresponding stimuli. We achieved this by adding a random deviation to the x and y coordinates of the fixations from a normal distribution, N(0, ε_xy_), where ε_xy_ is a free parameter described below. Next, we simulated how a poorly calibrated eye-tracker might capture those fixations. To do this, we distorted the entire pattern of fixations by multiplying a distortion matrix by all the fixation vectors. The distortion matrix was built by adding random values from a normal N(0, ε_D_) distribution to each element of a 2 × 2 identity matrix. The circles in the central panel of Fig. 2 show the result of applying this distortion to the original fixations. Finally, we tried to reconstruct the veridical pattern of fixations applying the function described in Listing 1 to the data depicted in the central panel. For the specific example represented in Fig. 2, the results are shown in the bottom panel.

Using this general procedure, we explored the impact of several parameters on the final quality of the data correction process. In our first simulation, we manipulated the number of fixations (1–10) keeping constant the number of stimuli on the screen (12). Parameter ε_xy_ was set to 30, which means that on average fixations tended to deviate 30 pixels (both in the *x* and in the *y* dimension) from the center of the stimuli they were directed at. Assuming that these data corresponded to a 520-mm width screen with resolution 1920 × 1200 and with participants seated at 65 cm, 35 pixels is roughly equivalent to 0.8° of visual angle. Note that setting ε_xy_ to 30 is a rather pessimistic assumption that limits the potential of the correction algorithm to improve the quality of data. We chose this value to test the performance of the algorithm in a difficult set of circumstances. Parameter ε_D_ was set to 0.03 because this resulted in an eye-tracker calibration error of around 1°, a value within the range of typical calibration errors found in eye-tracking experiments (Hansen & Ji, 2010). For each condition, we gathered data from 300 simulations, each one with different locations, different deviations sampled from N(0, ε_xy_), and different random values from the N(0, ε_D_) distribution added to the distortion matrix.

**Fig. 3:**
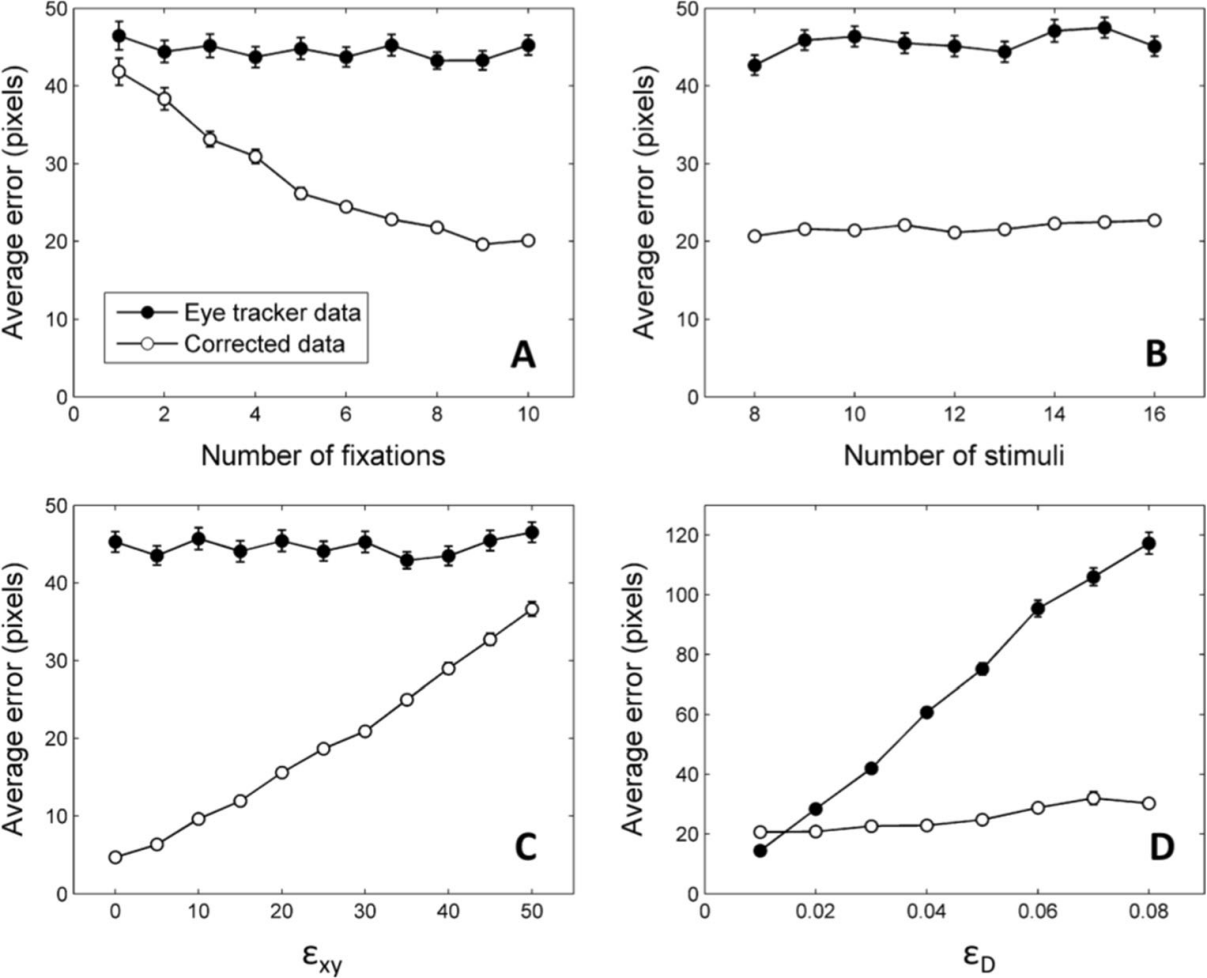
Results of four simulations testing the impact of four different parameters on the ability of the algorithm to reduce the error in the simulated eye-tracker data. The manipulated parameters are the number of fixations (**Panel A**), the number of stimuli on the screen (**Panel B**), parameter ε_xy_, representing the tendency of fixations to depart from the center of stimuli (**Panel C**), and parameter ε_D_, which determines the amount of calibration error (**Panel D**). Error bars denote standard error of the means across iterations

Figure 3A shows the average distance of the raw eye-tracker data and the corrected data to the veridical fixation coordinates. As can be seen, with these parameters the simulated eye-tracking data produced a consistent error of around 45 pixels. Assuming the above viewing distance, screen size, and resolution, these 45 pixels are roughly equivalent to 1° of visual angle. As mentioned above, this average error falls within the typical range found by researchers (Hansen & Ji, 2010; Hornof & Halverson, 2002; Johnson et al., 2007). Most interestingly, with these parameters, the correction algorithm reduces a substantial amount of the offset error found in the uncorrected data. The algorithm is able to utilize the information provided by the stimuli to provide a more accurate estimation of the true spatial pattern of the fixations. Furthermore, the quality of the corrected data increases considerably with the number of fixations. With seven or eight fixations, the algorithm is able to halve the amount of error, from approximately 45 pixels to 20–25 pixels (roughly 0.5°). Although these absolute values depend on the specific parameters of the simulation, these results show that the number of fixations produced by participants on each trial is an important criterion to keep in mind when deciding whether or not to use the correction algorithm.

Figure 3B shows the results of a similar simulation in which we explored the effects of manipulating the number of stimuli on the screen. For these simulations we used the same parameters and number of iterations as in the previous one. The number of simulated fixations was kept constant at eight and the number of stimuli on the screen had values from eight to 16. As can be seen, this manipulation had very little impact on the quality of the corrected data.

In the next simulation, we explored the impact of manipulating parameter ε_xy_. As explained above, our simulations assume that participants are not necessarily looking at the centers of the stimuli. Instead a random value from a N(0, ε_xy_) distribution is added to the x and the y coordinates of each fixation. As a result, larger values of ε_xy_ represent a larger tendency to make fixations that are far away from their corresponding stimulus. Given that the correction algorithm is based on the assumption that participants are looking at the stimuli, there are reasons to expect that its performance will be worse under conditions where real fixations depart from the centers of the stimuli. The results of our simulations, depicted in Fig. 3C, confirm these predictions. For these simulations ε_D_ was set to 0.03, the number of fixations was eight and the number of stimuli was 12. Parameter ε_xy_ had values ranging from 0 to 50. As expected, the ability of the correction algorithm to retrieve the correct fixation coordinates is compromised by the increasing tendency to look far away from stimuli. However, it is interesting to note that, even for large values of ε_xy_, the corrected data are still closer to the veridical coordinates of the stimuli than the uncorrected data. The relative success of the correction algorithm is probably due to the fact that in this simulation we included a relatively large number of fixations (eight) per display, which, as discussed above, improves the performance of the algorithm. This simulation shows that, at least when a large number of fixations are available, the algorithm is able to improve the quality of the data even when the average distance from fixations to stimuli is large. Most importantly, when ε_xy_ has very small values (0 or 5) the algorithm reduces the error to almost negligible levels, indicating that the correction procedure is particularly valuable for experimental paradigms in which participants’ fixations are well directed towards stimuli.

Finally, we explored the ability of the algorithm to retrieve the correct coordinates under different eye-tracker calibration conditions. As mentioned above, a poor calibration of the eye-tracker was simulated by multiplying the coordinate vector of each fixation by a distortion matrix. To build the distortion matrix, random values from a N(0, ε_D_) distribution were added to each element of a 2 × 2 identity matrix. The previous simulations were conducted with ε_D_ set to 0.03 because this value gave rise to calibration errors similar to those observed in real experiments (around 45 pixels, roughly 1° of visual angle). In the next simulation we manipulated ε_D_ with values ranging from 0.01 to 0.08. The number of simulated fixations was eight, with 12 stimuli per display and parameter ε_xy_ set to 30. The results of the simulation are shown in the bottom right panel of Fig. 3D. With very low values of ε_D_ the corrected data are no better than the uncorrected data. This result is hardly surprising: It is very difficult to improve the quality of data if the calibration of the eye-tracker is already very good. Note, however, that even for relatively low values of ε_D_ the corrected data are more accurate than the uncorrected data. When ε_D_ is equal to 0.02, the uncorrected data show an average error close to 30 pixels (around 0.7° of visual angle). This would be considered a relatively good calibration in many experimental paradigms (see Hansen & Ji, 2010, Fig. 9). However, the accuracy is even better for the corrected data. This means that even in situations where the calibration would be considered relatively good, the correction algorithm can still improve accuracy. As values of ε_D_ increase, the algorithm performs well in retrieving the correct coordinates, with greater calibration error (ε_D_) having only a very slight effect on the accuracy of the corrected data.

## The impact of random fixations

An important shortcoming of the previous simulations is that all of them assumed that fixations were somehow related to the coordinates of the stimuli presented on the screen. Setting parameter ε_xy_ to large values allowed us to explore the quality of the correction under conditions in which fixations tended to deviate markedly from their corresponding stimulus, yet each fixation was always directed to some stimulus with a degree of noise. It is important to consider alternative patterns of fixations, since this assumption may not hold in all conditions. For example, it is known that participants sometimes tend to fixate the center of the scene independently of the distribution of image features in scene viewing (Tatler, 2007). Additionally, saccades can undershoot and overshoot the distance to a target (e.g., Drieghe, Rayner & Pollatsek, 2008; Reilly & O’Regan, 1998). Fixations may even be purposefully allocated to the spaces between stimuli in order to gain a sense of the global array and relations between the items (Reingold et al., 2001). In principle, this poses a problem for all correction algorithms that aim to minimize the distance between each fixation and a specific stimulus (e,g., Hornof & Halverson, 2002; Zhang & Hornof, 2011). As such, researchers should bear in mind the context in which the fixations are made rather than blindly applying the algorithm to all fixation data.

As a means to explore the impact of these fixations on our correction algorithm, we ran a simulation in which an entirely random fixation was added to the stimulus-based set of fixations. Essentially, this was a replication of the simulation reported in Fig. 3A, only that the set of fixations now included an additional fixation directed to a completely random location on the screen. For this simulation, parameter ε_xy_ was set to 30, ε_D_ was equal to 0.03, the number of stimuli was 12, and the number of fixations was manipulated with values from one (only the random fixation) to 13 (the random fixation plus 12 fixations to stimuli).

**Fig. 4:**
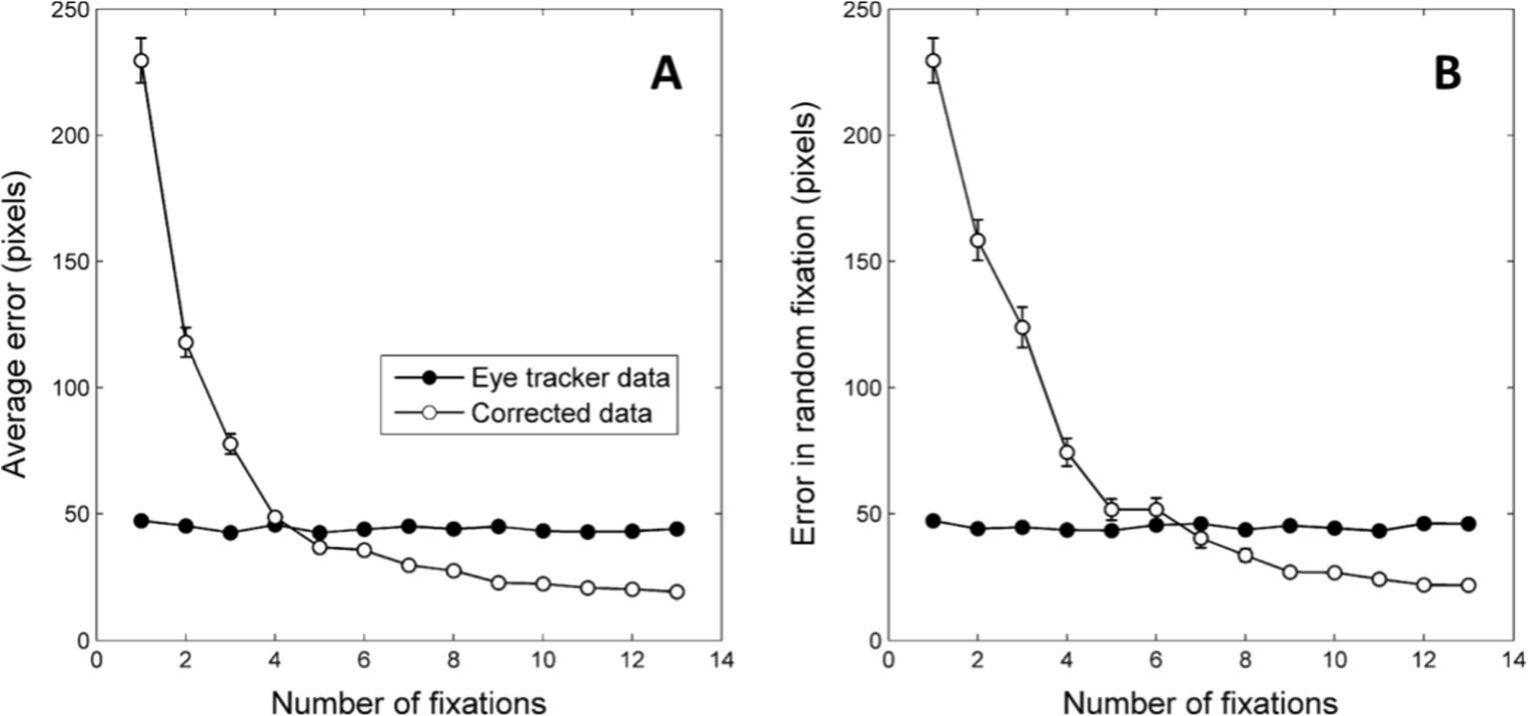
Results of a simulation exploring the impact of including a fixation to a random place on the screen among other fixations to non-random locations. The dependent variable in Panel A is the average distance between all recovered fixations (the random and the non-random ones) and the real location of fixations. In Panel B the error is computed only for the random fixation (ignoring the error of the non-random fixations). Error bars denote standard error of the means across iterations

The results of the simulation are shown in Fig. 4. Figure 4A depicts the same dependent variable used in previous simulations, namely the average distance of all (corrected and uncorrected) fixations to their real coordinates. Comparison of this simulation with the previous ones clearly shows that including a random fixation had a negative impact on the ability of the algorithm to reduce the calibration error. Not surprisingly, when the random fixation is the only one available, the correction algorithm increases the error dramatically. This is due to the fact that the algorithm adjusts the coordinates of the fixation by dragging the recorded fixation position towards the closest stimulus on the screen, which is often away from the true position of the fixation. However, when the data set not only includes the random fixation, but also fixations to stimuli, the accuracy of the corrected data increases progressively as more fixations are fed into the algorithm. With the specific parameters used in our simulation, the quality of the corrected data exceeds that of the uncorrected data with just five fixations (the random fixation plus four fixations towards stimuli). Note that these parameters include a tendency of all fixations to depart from their respective stimulus (ε_xy_ = 30). Therefore, the algorithm is able to correct eye-tracking data with just five fixations, even when one of them is directed towards an entirely random location on the screen and the other four reflect typical inaccuracies in the sampling of fixations to stimuli. With a sufficient number of fixations (e.g., ten), the negative impact of the random fixation becomes negligible.

To get a clearer idea of how the correction algorithm treats specifically the random fixation, we also computed the distance between the corrected coordinates for the random fixation and its real coordinates. In other words, we measured the ability of the algorithm to retrieve the correct location of the fixation that was not directed to a stimulus, ignoring how it fitted the fixations that were directed at stimuli. This information is shown in Fig. 4B. As can be seen, when sufficient fixations to stimuli are available, the algorithm provides a good correction even for the specific fixation that was directed to a random location. In other words, when enough stimulus-based fixations are included, random fixations are fitted to their veridical locations, instead of just being dragged to the closest stimulus.

## How to detect over-fitting

The previous simulation explored the impact of including a single random fixation in the quality of the resulting correction. In the worst-case scenario, participants might conduct the whole task by making fixations to random locations on the screen. Under those circumstances, the algorithm cannot correct for the calibration error because the pattern of stimuli does not provide any meaningful information for estimating the location at which participants might be fixating. In fact, the size of the (erroneous) corrections computed by the algorithm under these circumstances is so large that it is relatively easy to detect this kind of situation.

**Fig. 5:**
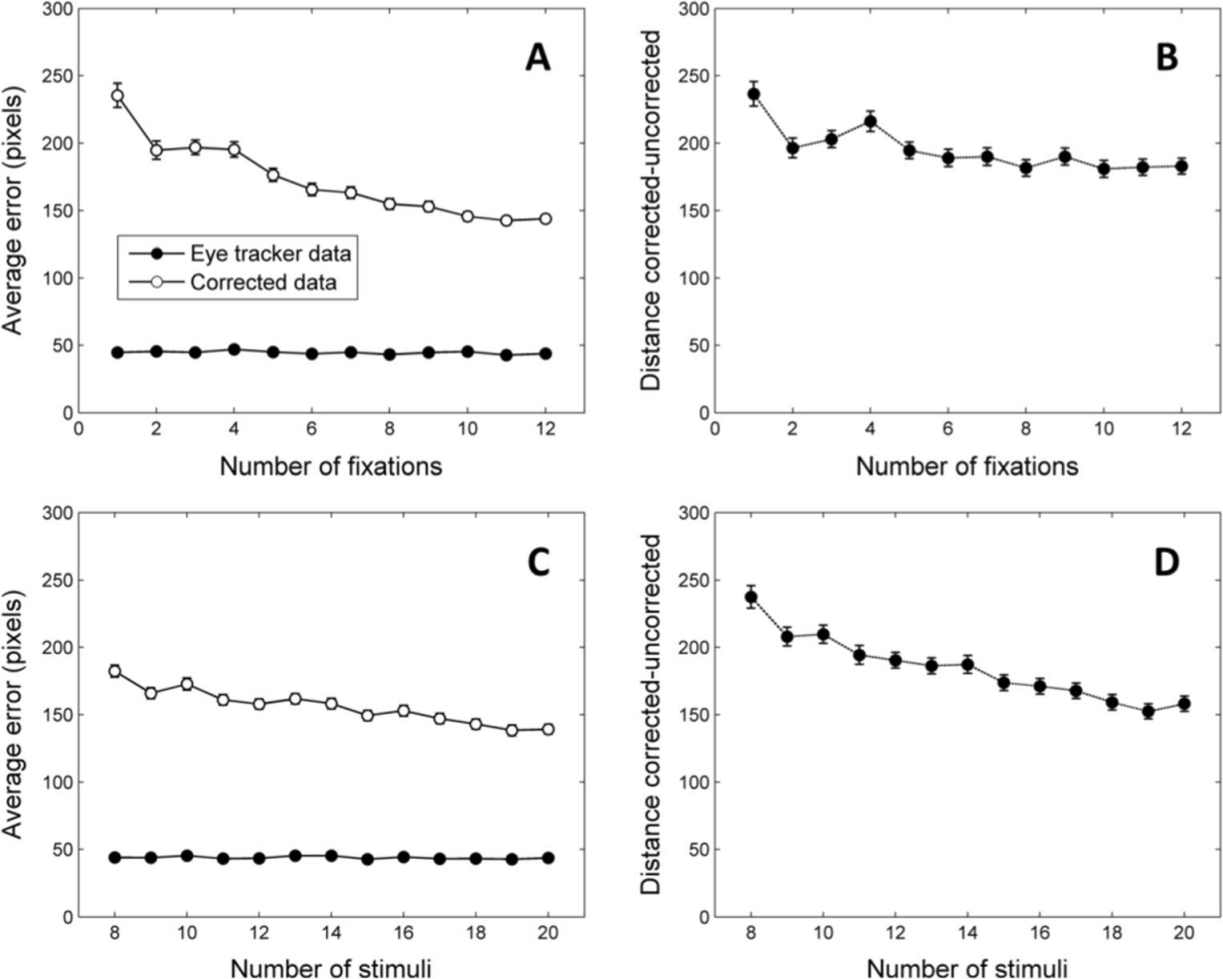
Results of two simulations exploring the performance of the algorithm when all fixations are directed towards random places on the screen. In the top panels, the number of fixations is manipulated keeping other parameters constant. In the bottom panels, the number of stimuli on the screen is manipulated. The left panels represent the average distance from the corrected and the uncorrected eye-tracker data to the real coordinates. The right panels show the average distance from the uncorrected coordinates reported by the eye-tracker and the coordinates retrieved by the algorithm. Error bars denote standard error of the means across iterations

Figure 5 depicts the results of two additional simulations that we conducted to explore this problem. In these simulations, all fixations were in random locations on the screen. Panels A and B show the results of manipulating the number of fixations, keeping the number of stimuli at 12. Panels C and D show the results of manipulating the number of stimuli, keeping the number of fixations constant at eight. In both cases ε_D_ was set to 0.03. As can be seen, under no circumstances are the corrected data better than the uncorrected data. As described above, this result is expected, since in the case of fixations directed towards random positions the application of a correction towards the location of the stimuli will lead to an increase in the deviation from the veridical positions of the fixations. Figure 5B and D represent the average distance between each corrected and each uncorrected data point. In all cases the corrected data depart from the original data, on average, by a distance of between 150 and 250 pixels. This would represent a correction of approximately 3.5°–5.7° of visual angle. This amount of correction is obviously much larger than the size of the errors one would expect to find in standard eye-trackers. A pattern of corrections like this would suggest either that the calibration of the eye-tracker was so unusually poor that the data are far too noisy to use for analysis or, more likely, that the pattern of fixations was unrelated to the stimuli presented on the screen.

## Correcting real data

The main advantage of testing the algorithm with simulated data, instead of real data, is that in the simulations it is possible to have privileged access to the veridical coordinates of the simulated fixations (i.e., the left-most column in Fig. 2). This valuable piece of information is missing in the case of real data, where only the data provided by the eye-tracker (represented in the central column of Fig. 2) are available. However, relying on simulated data has a major disadvantage: The evaluation is valid only if the simulated pattern of error reflects the characteristics of natural eye-tracking error. So far, we have assumed that the eye-tracking instrumentation error can be simulated through a 2 × 2 distortion matrix. Although this is a plausible assumption in many cases, it is known that eye-tracking error can be non-linear (Cerrolaza, Villanueva, & Cabeza, 2012; Drewes, Masson, & Montagnini, 2012) and that quadratic functions are sometimes needed to correct these sources of error (Zhang & Hornof, 2014). Since our previous simulations ignored these potential sources of calibration error, the following analyses explored the algorithm’s ability to correct real eye-tracking data.

For the sake of brevity, the full details of the design, procedure, and results are not reported. In brief, 20 participants were exposed to a series of 192 trials. In each of these trials, a 1-s fixation cross was followed by a search display like the ones depicted in Fig. 1. Participants were instructed to find a T-shaped target among a number of similar distractors and report the orientation of the T by pressing <Z> if the stem of the T pointed towards the left and <M> if it pointed towards the right. After pressing the correct button, all the stimuli disappeared from the screen and the next trial began 1 s later. If participants pressed the wrong key, an error message was presented for 3 s before proceeding to the 1-s inter-trial interval.

Eye movements were recorded using a head-mounted EyeLink II system. The eye-tracker was calibrated at the beginning of the experiment using the manufacturer’s calibration routine with standard settings. The EyeLink II calibration routine asks participants to look sequentially at nine points on the screen. The system uses the data gathered during this calibration to fit a model that matches ocular parameters with the known locations of the nine points. Immediately afterwards, the calibration is validated by asking the participant again to look at a similar nine-point grid. This calibration process was repeated as many times as needed until the EyeLink system reported an accuracy of 0.5° or less. The entire calibration process was repeated again in the middle of the experiment. In addition to these calibrations, we conducted a drift correction every 12 trials using the EyeLink II system’s built-in routine. This routine simply asks participants to fixate on a small point presented at the center of the screen. If there is a discrepancy between the point where the participant seems to be looking and the coordinates of the center of the screen, this disparity is used to correct the calibration during the subsequent trials. After collecting the data, all fixations were corrected offline with the algorithm presented in Listing 1. Because when the trial began some participants were still looking at the place where the fixation cross had been, we entered the fixation cross as an additional stimulus.

**Fig. 6:**
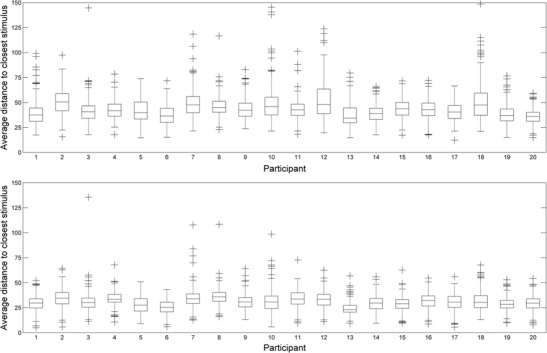
Average distance (in pixels) from fixations to the closest stimulus across trials for each participant. The two panels represent the average distances before (top) and after (bottom) applying the error-correction algorithm

Figure 6 shows the average distance of fixations from their closest stimulus across trials for each participant. The top and bottom panels represent the average distances before and after the correction, respectively. As can be seen, before applying the error-correction algorithm, most fixations tended to be within the range of 30 (0.7°) to 60 pixels (1.4°) away from stimuli. After clearning up the data with the algorithm, most of the corrected fixations were in the range of 25 (0.6°) to 35 pixels (0.8°). The mean average distance changed from 43.61 pixels (*SD* = 5.16) before the correction, to 30.86 pixels (*SD* = 3.09) after the correction.

Does this reduction represent a genuine correction of calibration errors? Given the details of the experimental task, there are known locations where the participant is very likely to fixate at specific moments in the task. For instance, given that participants are instructed to find the target and that the trial ends when they report its orientation, it is likely that the last fixation registered in each trial will usually (although possibly not always) be directed towards the target. Consistent with this, the last fixation on each trial was on average only 48 pixels (1.1°) away from the target location. Most importantly, after applying the error-correction algorithm, this distance was reduced to 35 pixels (0.8°). This difference was significant and demonstrated a large effect size, *t*(19) = 9.25, *p* < .001, *d*_*z*_ = 2.06. This result strongly suggests that the algorithm is correcting a real calibration problem.

**Fig. 7:**
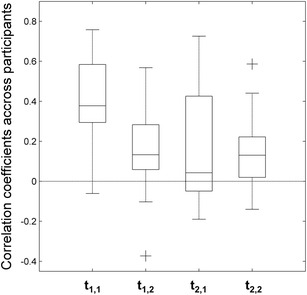
Mean correlation between the parameters needed to optimize fixations during the first six trials and the last six trials of each block. Variables t_1,1_, t_1,2_, t_2,1_, and t_2,2_ represent the four values in the best-fitting transformation matrix

It is also possible to check the validity of the algorithm in real data by exploring to what extent the corrections made by the fitting process are plausible. For instance, in real settings, one would usually expect the calibration of the eye-tracker to degrade slowly over time (e.g., the position of the participant’s head with respect to the eye-tracker may undergo subtle changes). Therefore, the transformations conducted on the data from a set of trials should be correlated with the transformations conducted on adjacent trials. In our experiment, drift was corrected every 12 trials. As a result, we would expect the calibration to remain relatively constant within each of these blocks of trials. Therefore, the transformation matrix used by the algorithm to correct data from one of these trials should not differ greatly from the transformation matrix used to correct the rest of the trials within the block. To confirm this prediction, we averaged the values of the transformation matrix across the first six trials of each block and across the last six trials of each block. We then computed the correlation coefficient of those two measures across blocks for each participant. Figure 7 depicts the aggregated data of all participants for each of the four entries of the transformation matrix T, denoted by t_1,1_, t_1,2_, t_2,1_ and t_2,2_. For the four entries, the mean correlation between the first and the last trials of each block was significantly different from zero with a medium to large effect size, smallest *t*(19) = 2.85, *p* = .01, *d*_*z*_ = 0.64.

This result is interesting for two reasons. Firstly, the fact that the best-fitting transformation matrices remain similar across consecutive trials shows that the algorithm is correcting a stable source of noise in the data and not just providing a random over-fitting of the fixations. Secondly, there can be no covariation without variation. The values of the transformation matrices used in adjacent sub-blocks of trials covaried across the experiment because there was variation in them. In other words, the transformation matrices that worked best to correct calibration problems at some moments in the experiment were not the best ones for other moments. Note that this combination of results (similar transformation matrices in consecutive trials, but different matrices across the experiment) would never have arisen if the calibration of the eye-tracker had been perfect or if the drift corrections conducted every eight trials had sufficed to correct for calibration problems. This suggests that calibration quality did actually change over time, confirming the need to use some method for the offline calibration of data.

## Concluding remarks and recommendations

In the previous sections, we have outlined the basic properties of a simple procedure for the offline recalibration of eye-tracking data. As shown in Listing 1, the essential part of the procedure can be easily implemented in a brief script. For the sake of brevity, our analyses and simulations have focused on that simplified version of the algorithm. However, as the astute reader may have noticed, this procedure can be modified in different ways to improve its performance in particular settings.

For instance, the main conclusion of the simulations is that the algorithm works best when a relatively large number of fixations are included. When this condition is met, the algorithm can deal successfully with fixations that are not close to their respective stimuli (Fig. 3C), extremely poor calibrations (Fig. 3D), and even with some fixations in random locations (Fig. 4). The results are not so good when the fitting process is based on only a small number of fixations. However, a minor change in the procedure will allow this algorithm to be applied to situations that give rise to a small number of fixations per trial. Our data show that fixations on adjacent trials are likely to require a similar correction. Based on this idea, it is possible to correct data from one trial using information about the fixations and stimuli of the adjacent trials as well. Collapsing data from many trials in this way permits the use of the present algorithm to improve the accuracy of data even in experimental procedures that elicit only two or three fixations per trial.

Similarly, it is possible to adapt the basic procedure to experimental settings where fixations at random locations of the screen are frequent. Under normal circumstances, many eye-trackers can be expected to have a calibration error of 1° of visual angle. Therefore, if a fixation is more than 1° or 2° away from its closest stimulus, that fixation is relatively unlikely to be directed towards any stimulus. Based on this idea, the quality of the correction can be improved by removing from the algorithm’s input all the fixations that are unusually far away from their closest stimulus. We have found that 3–4° reflects a relatively safe and liberal threshold criterion. This change can be easily implemented in the script described in Listing 1 by changing Line 10, so that only distances lower than a given threshold are stored and taken into account.

As mentioned above, an important limitation of the present algorithm is that it cannot correct for non-linear sources of eye-tracking error (Cerrolaza et al., 2012; Drewes et al., 2012). If researchers have reasons to suspect that their data are substantially affected by non-linear error, then it might be preferable to resort to correction procedures that include quadratic or other non-linear functions (Zhang & Hornof, 2014). Similarly, the present algorithm does not allow the correction of errors that require a rotation of the space of fixations. To correct for such errors, it is necessary to fit a rotation matrix with trigonometric functions (Johnson et al., 2007). Note, however, that the experienced reader can modify our algorithm easily to include these more sophisticated corrections. For instance, the  function in Listing 1 can be easily modified to take as its argument not only a transformation matrix, but also a rotation matrix, whose parameters can be fitted using the same general procedure. This would require only a minor modification of lines 2–5 in Listing 1. A similar approach can be taken to correct for non-linear errors.

It is important to note, however, that these potential extensions of the algorithm come at a cost: The flexibility of these extensions not only allows them to correct for additional sources of error, but also increases the risk of overfitting. This is particularly likely to happen if the correction is not constrained by a sufficiently large number of data points. For instance, the method proposed by Zhang and Hornof (2014) to correct for non-linear error involves fitting 12 coefficients, instead of the four coefficients fitted in the algorithm that we describe in this paper. This difference in the number of free parameters allows Zhang and Hornof’s (2014) method to correct for sources of error that our algorithm cannot accommodate. But it also increases substantially the risk of overfitting. The scale of this problem is difficult to assess in the absence of a thorough exploration like the one conducted in the present study. Future research should explore the robustness of these non-linear correction procedures in the face of overfitting and provide specific guidelines for limiting the potential impact of excessive flexibility.

In our simulations and analyses of real data we have only considered experimental settings in which the locations of stimuli are objectively and clearly defined, such as finding a target among a number of similar distractors appearing on a homogeneous background. In principle it is also possible to apply this algorithm to scene viewing studies and other situations where stimuli and regions of interest may be less well defined (e.g., Brockmole & Henderson, 2006), by assuming that there are specific parts of the display where the participant is very likely to fixate. For example, if there are some regions with high contrast or with a high salience, it would be possible to fit a transformation matrix that minimizes the distance between fixations and those locations. It would also be possible to diagnose whether the resulting corrections are valid by checking whether they are relatively consistent over trials. That is, if the transformation matrix undergoes considerable variation across adjacent trials, it would suggest that the experimenter-defined regions of interest do not map onto the locations that participants fixated.

In summary, the algorithm we have described is able to considerably improve the validity of eye-tracking data as shown in the simulations and the recalibration of real data. Furthermore we have described ways to improve the performance of the algorithm in experimental settings that elicit just a few fixations per trial, that give rise to many fixations at random places or to stimuli that do not fall in clearly defined regions of interest.
